# Antibiotic Resistance in *Campylobacter* spp. Isolated from Broiler Chicken Meat and Human Patients in Estonia

**DOI:** 10.3390/microorganisms10051067

**Published:** 2022-05-22

**Authors:** Triin Tedersoo, Mati Roasto, Mihkel Mäesaar, Liidia Häkkinen, Veljo Kisand, Marina Ivanova, Marikki Heidi Valli, Kadrin Meremäe

**Affiliations:** 1 Chair of Food Hygiene and Veterinary Public Health, Institute of Veterinary Medicine and Animal Sciences, Estonian University of Life Sciences, Kreutzwaldi 56/3, 51014 Tartu, Estonia; mati.roasto@emu.ee (M.R.); mihkel.maesaar@emu.ee (M.M.); marikki.valli@gmail.com (M.H.V.); kadrin.meremae@emu.ee (K.M.); 2 Veterinary and Food Laboratory, Kreutzwaldi 30, 51006 Tartu, Estonia; liidia.hakkinen@vetlab.ee; 3 Institute of Technology, University of Tartu, Nooruse 1, 50411 Tartu, Estonia; veljo.kisand@ut.ee; 4 Central Laboratory, East-Tallinn Central Hospital, Ravi 18, 10138 Tallinn, Estonia; marina.ivanova@itk.ee

**Keywords:** *Campylobacter* spp., antibiotic resistance, multidrug resistance, fresh broiler chicken meat, resistance genes

## Abstract

Poultry meat is considered the most important source of *Campylobacter* spp. Because of rising antimicrobial resistance in *Campylobacter* spp., this study investigated the antimicrobial resistance of *Campylobacter* isolates from fresh broiler chicken meat originating from the Baltic countries sold in Estonian retail settings. Additionally, human clinical isolates obtained from patients with *Campylobacter* enteritis in Estonia were analysed. The aim of this study was to investigate the susceptibility of *Campylobacter* spp. to nalidixic acid, ciprofloxacin, tetracycline, streptomycin, erythromycin and gentamicin. The broth microdilution method with the EUCAMP2 panel was used for MIC determination and antimicrobial mechanisms were analysed using WGS data. A total of 46 *Campylobacter* strains were analysed, of which 26 (42.6%) originated from Lithuanian, 16 (26.2%) from Latvian, and 4 (6.6%) from Estonian fresh broiler chicken meat. In addition, 15 (24.6%) *Campylobacter* strains of patients with campylobacteriosis were tested. The antimicrobial resistance patterns of *Campylobacter* spp. isolated from fresh broiler chicken meat samples of Estonian, Latvian and Lithuanian origin collected in Estonian retail, and from patients with *Campylobacter* enteric infections, were determined. A total of 46 (75%) of the isolates tested were *C. jejuni* and 15 (25%) were *C. coli*. *Campylobacter* resistance was highest to nalidixic acid (90.2% of strains) and ciprofloxacin (90.2%), followed by tetracycline (57.4%), streptomycin (42.6%) and erythromycin (6.6%). All strains were sensitive to gentamicin. Additionally, antimicrobial resistance genes and point mutations were detected in 27 *C. jejuni* and 8 *C. coli* isolates previously assigned as resistant with the phenotypic method. A high antibiotic resistance of *Campylobacter* spp. in Lithuanian- and Latvian-origin broiler chicken meat and Estonian clinical isolates was found. Similar antibiotic resistance patterns were found for broiler chicken meat and human *Campylobacter* isolates.

## 1. Introduction

*Campylobacter* is small spiral Gram-negative bacterial pathogen and the main agent of campylobacteriosis, which is the most common zoonotic disease in the EU transmitted to humans directly from animals, or through the foodborne route [1,2]. Recent source attribution analysis has revealed the prevalent role of broiler chickens as the cause of human *Campylobacter* infections in the Baltic countries [3]. Broilers and broiler chicken meat are the most important sources of *Campylobacter* infections in humans [4,5,6]. Disease caused by *Campylobacter* spp. is generally mild and self-limited. Nevertheless, it can cause severe systematic infection in children/elderly people and humans with immunosuppression, and also—in very rare cases—Guillan-Barré syndrome [7,8]. Such occasions often require therapy with first-line antibiotics such as fluoroquinolones, e.g., ciprofloxacin, and macrolides, e.g., erythromycin [9,10,11,12,13,14]. Over time, *Campylobacter* has acquired resistance to these antibiotics which are considered critically important for the treatment of *Campylobacter* infections [11,15,16]. The increasing resistance of thermophilic *Campylobacter* spp. to antibiotics could lead to detrimental effects on public health [2,17,18,19]; therefore, the World Health Organization has classified many of these antimicrobials as critically important for human medicine [20].

Previous studies [21,22] have identified different levels of *Campylobacter*-contaminated poultry meat in the Baltic states, and the proportion of antimicrobial-resistant *Campylobacter* spp. strains in poultry meat has been found to be very high in both Lithuania and Latvia [23,24]. This is affecting public health and needs to be addressed.

This study aimed to determine the proportions of antimicrobial-resistant *Campylobacter* strains from fresh broiler chicken meat of Estonian, Latvian and Lithuanian origin at the Estonian retail level, and among strains of human clinical infections. Additionally, resistance pheno- and genotypes were determined and compared. The results of this work will make it possible to assess the trends in antimicrobial resistance over a long period and determine related public health risks.

## 2. Materials and Methods

### 2.1. Campylobacter Isolates

In total, 61 *Campylobacter* isolates were studied, of which 46 (75%) were *C. jejuni* and 15 (25%) *C. coli*. From the broiler chicken meat isolates, 34 (73.9%) were *C. jejuni* and 12 were (26.1%) *C. coli*. Among the clinical isolates, 12 (80%) and 3 (20%) were *C. jejuni* and *C. coli*, respectively. All isolates were obtained from a previous study on *Campylobacter* spp. in Estonia [25]. The samples consisted of all *Campylobacter* strains isolated from Estonian (n = 4), Latvian (n = 16) and Lithuanian (n = 26) fresh broiler chicken meat products in Estonian retail from our previous study [25]. Additionally, isolates from human patients (n = 15) originating from ambulatory and hospitalized patients from north and north-eastern Estonia were obtained from East-Tallinn Central Hospital and Rakvere Hospital were included. *Campylobacter* detection from broiler chicken meat samples was performed according to ISO 10272-1:2017 [26], as described by Tedersoo et al. [25]. In brief, for *Campylobacter* spp. detection, 10 g of broiler chicken meat sample was transferred into 90 mL Preston enrichment broth and incubated in microaerobic conditions at 41.5 ± 0.5 °C for 24 h. Then, a 10 µ loopful of Preston enrichment broth was inoculated onto mCCD agar medium (Oxoid Ltd.; Basingstoke, Hampshire, UK). All plates were incubated in microaerobic conditions at 41.5 °C ± 0. 5 °C for 48 h. Typical *Campylobacter* colonies on mCCDA plates were streaked on Columbia blood agar (Oxoid Ltd.; Basingstoke, Hampshire, UK), which were incubated for 24 h at 41.5 °C ± 0.5 °C. Additional confirmation tests included Gram staining, motility analysis, and oxidase and catalase tests were performed.

### 2.2. Antimicrobial Susceptibility Testing

Minimal inhibitory concentration (MIC) values for nalidixic acid, ciprofloxacin, tetracycline, streptomycin, erythromycin and gentamicin were determined by using the broth microdilution method with the EUCAMP2 panel (TREK diagnostic Systems Ltd., East Grinstead, UK) in accordance with the manufacturer’s protocols.

The cut-off values recommended by the European Committee on Antimicrobial Susceptibility Testing were used for *C. jejuni* and *C. coli* in accordance with the European Commission implementing decision 2013/652/EU on the monitoring and the reporting of antimicrobial resistance in zoonotic and commensal bacteria. *C. jejuni* was assigned resistant when MIC values equated to: erythromycin > 4 µg/mL, ciprofloxacin > 0.5 µg/mL, tetracycline > 1 µg/mL, streptomycin > 4 µg/mL, nalidixic acid > 16 µg/mL or gentamicin > 2 µg/mL. *C. coli* was assigned resistant when MIC values equated to: erythromycin > 8 µg/mL, ciprofloxacin > 0.5 µg/mL, tetracycline > 2 µg/mL, streptomycin > 4 µg/mL, nalidixic acid > 16 µg/mL or gentamicin > 2 µg/mL.

Analyses were performed in the Veterinary and Food Laboratory of Estonia, which is also the national reference laboratory.

### 2.3. Whole-Genome Sequencing and Analysis of Resistant Genes

Molecular analysis, whole-genome sequencing and bioinformatics were performed as described by Tedersoo et al. [25]. In brief, the sequencing was carried out on an Illumina NextSeq500 System (Illumina, Inc.; San Diego, CA, USA) using the NextSeq 500/550 High Output Kit v2.5 (300 Cycles) in paired-end 2 × 151 bp mode. All genome sequences were submitted to the *C. jejuni*/*C. coli* multilocus sequence typing (MLST) database [27].

Antimicrobial resistance genes and point mutations of *C. jejuni* (n = 27) and *C. coli* (n = 8) isolates previously assigned as resistant with the MIC test were detected in the subset of isolates. MIC-sensitive campylobacters were not included in the analysis and only genotypic resistance mechanisms corresponding to phenotypic AMR were identified and reported in present study. AMRFinderPlus v3.10.23 with database v2021-12-21.1 (downloaded 3 March 2022) was used according to the default settings, except for the organism “*Campylobacter*” and the “plus” options [28,29]. Genes with coverage of less than 80% were not included in the analysis.

### 2.4. Statistical Analysis

MS Excel 2010 software (Microsoft Corporation; Redmond, WA, USA) was used to record the results. The Chi-squared test was used to test for statistically significant associations between the antimicrobial resistance of *Campylobacter* spp. in fresh broiler chicken meat from different sources. The results were considered statistically significant for *p* values of ≤0.05.

## 3. Results

The results showed that a total of six (9.8%) isolates were sensitive to all the tested antibiotics: four isolates from Estonian-origin chicken meat and two human *Campylobacter* strains. *Campylobacter* isolates of broiler chicken meat origin showed the highest resistance to quinolones, tetracycline and streptomycin. In addition, clinical *Campylobacter* isolates were found to be most resistant against the same antibiotics. All *Campylobacter* strains were sensitive to gentamicin (Table 1). Significant differences (*p* < 0.001) were found in nalidixic acid, ciprofloxacin and tetracycline resistance among Estonian versus Latvian and Lithuanian *Campylobacter* isolates from fresh broiler chicken meat. The Estonian broiler-chicken-meat-origin *Campylobacter* isolates were significantly (*p* < 0.001) less resistant to fluoroquinolones than those strains which originated from Latvian and Lithuanian broiler chicken meat and Estonian human patients. There were no differences detected in streptomycin, erythromycin or gentamicin resistance between *Campylobacter* broiler chicken meat isolates of Estonian versus Latvian and Lithuanian origin. Resistance in the human isolates and broiler chicken meat isolates of Latvian (*p* = 0.13) and Lithuanian (*p* = 0.06) origin did not differ significantly. A total of 55 (90.2%) isolates were resistant to one or more antibiotics: 10 (16.4%) were resistant to one antibiotic, 28 (45.9%) were resistant to at least two antibiotics not belonging to the same group of antimicrobials (fluoroqinolones and quinolone (ciprofloxacin, nalidixic acid), macrolides (erythromycin), tetracyclines (tetracycline) and aminoglycosides (streptomycin, gentamicin)), and 17 (27.9%) isolates were resistant to three or more antibiotics not belonging to the same group. The proportion of isolates resistant to *C. jejuni* and *C. coli* was 87% and 100%, respectively. Antimicrobial resistance to one or more antimicrobial was significantly higher (*p* < 0.001) in the *Campylobacter* isolates from the broiler chicken meat of Latvian and Lithuanian origin compared to that of Estonian origin. It was found that 27.9% of isolates were multidrug-resistant, of which 11 isolates (18.0%) were of Lithuanian and 2 (3.3%) of Latvian broiler chicken meat origin, and 4 (6.6%) were from Estonian human patients. Multidrug resistance was defined as resistance to three or more antibiotics not belonging to the same group. All Latvian and Lithuanian isolates originating from broiler chicken meat were resistant to fluoroquinolones.

The resistance phenotypes of *Campylobacter* isolates are presented in Table 2. The most prevalent antimicrobial resistance pattern was Cip/Nal/Tet, with 55.3% and 21.7% in human and chicken meat isolates, respectively. Other common resistance phenotypes were Cip/Nal/Tet/Str and Cip/Nal/Str.

MIC values of *Campylobacter* are shown in Table 3. Very high minimum inhibitory concentrations were found for four erythromycin-resistant, 26 ciprofloxacin-resistant, 31 tetracycline-resistant, 51 nalidixic acid-resistant and 26 streptomycin-resistant *Campylobacter* isolates with MIC values ≥ 128 µg/mL, ≥16 g/mL, ≥64 µg/mL, ≥64 µg/mL and ≥16 µg/mL, respectively.

Altogether, 28 *C. jejuni* and 7 *C. coli* isolates, of which 29 were of broiler chicken meat origin and 6 were of human origin, previously assigned as resistant with the MIC test, were sequenced and all their antimicrobial resistance genes and point mutations are presented in Table 4. In total, 29 *Campylobacter* isolates from broiler chicken meat and 6 *Campylobacter* isolates of human origin showed resistance to quinolones, and all contained a point mutation T86I in the *gyrA* gene. The genotypic antibiotic resistance against tetracyclines (*tetO*) was 62% and 83% in broiler chicken meat (n = 18) and human isolates (n = 5), respectively. A total of 52% of broiler chicken meat isolates (n = 15) showed resistance against aminoglycosides and macrolides. The resistance against macrolides in human isolates was 50% (n = 3).

## 4. Discussion

As stated in the European Union Regulation No. 1831/2003, antimicrobials as growth promoters in food animal production have been banned since 2006 [30]. Antimicrobials are still used intensively in poultry for therapy and infection prophylaxis, which has caused the spread of resistant strains to humans [31,32]. However, some countries are showing positive trends, for example, antimicrobial use in poultry in Scandinavian countries is generally low. Denmark has declared carbapenems, third- and fourth-generation cephalosporins, fluoroquinolones and colistin as ‘critically important’, and the use of these antimicrobials is restricted [33]. Based on the report of DANMAP [33], cephalosporins and colistin are not used in Danish poultry production and the use of fluoroquinolones is close to zero.

The situation in Swedish broiler production is very good since the use of antibiotics is infrequent [34]. Consequently, the prevalence of resistant bacteria isolated from animals in Sweden is low [35,36].

FINRES-Vet [37] reports that the occurrence of antibiotic-resistant *Campylobacter* spp. from broilers has been at a low level. Compared to previous years, in 2020, the proportion of quinolone-resistant isolates dropped and the resistance to tetracycline, erythromycin, gentamicin or streptomycin remained low.

The annual NORM-VET 2020 report showed improvements of antimicrobial resistance in Norway [38]. As stated in this report, the prevalence of antimicrobial resistance among *C. jejuni* isolates from broilers is low; 90.8% of isolates were susceptible to all tested antimicrobials. Although the isolates were commonly resistant to quinolones and streptomycins, there were no multidrug-resistant isolates detected [38].

The rapid spread of antimicrobial resistance has been identified across the world and it is associated with the use of antimicrobials [39]. According to the European Food Safety Authority and the European Centre for Disease Prevention and Control (EFSA and ECDC) [40], in 2019, ciprofloxacin resistance in human *Campylobacter* isolates was high to extremely high (at the EU level it was 61.5% and 61.2% for *C. jejuni* and *C. coli*, respectively). The resistance to erythromycin was low (1.5% and 12.9% for *C. jejuni* and *C. coli*, respectively). *C. coli* erythromycin resistance was extremely high in Portugal (73.1%). The tetracycline resistance proportions were 47.2% and 66.9% for *C. jejuni* and *C. coli*, respectively [40]. According to EFSA and ECDC [40], the resistance to gentamycin in 2019 was low. In China, the prevalence of resistance in *Campylobacter* from human patients to ciprofloxacin, tetracycline and nalidixic acid is very high (89.7%, 74.6% and 69.0%, respectively), due to the extensive use of these antimicrobials without prescription [41]. In Ireland, compared to the early 2000s, tetracycline resistance among *Campylobacter* spp. in broilers has risen by approximately 10% [42]. In Portugal and Spain, *Campylobacter* spp. resistance to tetracycline in broilers is high: between 90 and 100% [43,44]. In a study conducted in Lithuania, Aksomaitiene et al. [23] found that *C. jejuni* isolates from human clinical cases were most frequently resistant to ciprofloxacin (88.1%), but all human isolates were sensitive to gentamicin and erythromycin. In our study, all the Estonian-origin broiler chicken meat *Campylobacter* isolates (n = 4) were sensitive to all of the studied antimicrobials. The small number of isolates was related to the very low *Campylobacter* prevalence (1.8% from 163 samples) in Estonian-origin broiler chicken meat [25]. The most frequently observed resistance (86.7%) of human strains was against ciprofloxacin and nalidixic acid. This high antimicrobial resistance among human strains probably indicates that ciprofloxacin and nalidixic acid would not be suitable for human *Campylobacter* infection treatment. In Estonia, the first choice of antibiotic treatment for human patients with severe *Campylobacter* infection is azithromycin, followed by ciprofloxacin as the alternative choice. In the present study, the proportions of *Campylobacter* isolates from fresh broiler chicken meat that were resistant to ciprofloxacin and erythromycin, all of Latvian and Lithuanian origin, were 91.3% and 8.7%, respectively. According to the EFSA and ECDC [40], in 2019, the highest levels of resistance in broiler meat were for nalidixic acid and ciprofloxacin (64–90%), and also for tetracycline (43–53%). A previous Estonian study by Mäesaar et al. [45] found similarly high proportions of fluoroquinolone resistance among Latvian (87.5%) and Lithuanian (84.8%) *Campylobacter* isolates originating from broiler chicken meat. In the present study, fluoroquinolone resistance of Latvian and Lithuanian isolates originating from broiler chicken meat was 100%, which probably reflects the wide use of these antibiotics in poultry production in these countries. The use of synthetic fluoroquinolone (enrofloxacin) for the treatment of respiratory and gastrointestinal infections in poultry has been shown to induce fluoroquinolone resistance in *Campylobacter* spp. [46]. Similarly to the results of the present study, Aksomaitiene et al. [23] found that all *C. jejuni* isolates from broiler products from Lithuanian retail settings were resistant to ciprofloxacin. Meistere et al. [24] reported that Latvia has one of the highest proportions of fluoroquinolone resistance among *C. jejuni* (97.5%) in broilers. Furthermore, Kovalenko et al. [47] found a high proportion of *Campylobacter* isolates from Latvian broilers resistant to fluoroquinolones (100%), ciprofloxacin (100%), nalidixic acid (87.9%) and streptomycin (39.6%). In the present study, fluoroquinolone resistance among human isolates was 86.7%, and 91.3% in broiler meat. Mäesaar et al. [45] found resistance to fluoroquinolones to be higher for humans (67.9%) than for broilers (60.2%). Multidrug-resistant strains were co-resistant to nalidixic acid and ciprofloxacin. Several studies in Canada and the USA have reported *Campylobacter* spp. ciprofloxacin resistance in up to 47% of *Campylobacter* strains [18,48,49]. In addition to high fluoroquinolone resistance among broiler chicken meat isolates, the present study observed high tetracycline resistance among Lithuanian broiler chicken meat isolates (76.9%) and high streptomycin resistance among Latvian and Lithuanian broiler chicken meat isolates: 68.8% and 42.3%, respectively. Tetracycline resistance among human isolates was 80.0%, which matched with the tetracycline resistance found among Lithuanian broiler chicken meat isolates (76.9%). In Table 4, the phenotypic resistance pattern and related genotypic mechanisms (gene, point mutation) are shown. All phenotypic resistance found for aminoglycosides, macrolides, quinolones and tetracyclines determined with the MIC test had corresponding genotypic antibiotic resistance mechanisms. In previous studies, *aad9, aadE*, *aadE-Cc* and *aph(3′)-IIIa* resistance genes [50,51,52,53] associated with aminoglycoside resistance were detected from all isolates with corresponding phenotypic resistance. For tetracycline resistance only, the *tetO* [54] gene was detected from isolates showing phenotypic resistance to tetracycline in the MIC test. The latter has also been found in several other studies [54]. Two point mutations in *23S* (A2075G) and *gyrA* (T86I) genes associated with erythromycin and quinolone resistance [52,55] in campylobacters were also found in our study. In addition, a previous study conducted in Estonia found *gyrA* (T86I) mutation in quinolone-resistant *C. jejuni* ST5 isolates [56]. Additionally, 50*S* ribosomal protein L22 modification (A103V) [31] was detected in 14 isolates with no corresponding phenotypic erythromycin (macrolide) resistance found. The majority of isolates with matching geno- and phenotypic resistance had high MIC, and often exceeded the maximum concentration ranges.

High *Campylobacter* resistance in chicken meat can be a key risk factor for the treatment of severe human campylobacteriosis cases in Estonia. The high proportions of resistance and similar antimicrobial pheno- and genotypes found from imported broiler chicken meat products and for Estonian human clinical isolates indicate that the consumption of imported broiler chicken meat might pose the risk of *Campylobacter* to the Estonian population.

The application of a vertically integrated management system and strict biosecurity and biosafety measures at all levels of broiler chicken production may be the reason for the very low *Campylobacter* prevalence and counts as well as low antimicrobial resistance among *Campylobacter* strains isolated from the Estonian-origin fresh broiler chicken meat.

## 5. Conclusions

Among the *Campylobacter* strains isolated from broiler meat in 2018–2019, a total of 90.2% were resistant to one or more kind of antibiotics. Multidrug resistance was found in 27.9% of isolates. *Campylobacter* isolates from Estonian fresh chicken meat were sensitive to all of the tested antibiotics. Isolates of Latvian and Lithuanian origin were 100% resistant to one or more of the antibiotics, and 86.7% of the Estonian human strains were resistant to one or more of the antibiotics. There was high antibiotic resistance in *Campylobacter* spp. in Lithuanian and Latvian isolates from fresh broiler chicken meat in the Estonian retail market. There was also a high antibiotic resistance in *Campylobacter* spp. of human origin. This suggests that broiler chicken meat poses a potential risk to humans as it is well known that broiler chicken meat is a main source of human campylobacteriosis. To minimize the emergence of *Campylobacter* resistance, it is crucially important to follow common policies and implement good practices on antimicrobial usage at the farm level. Resistant bacteria in the food production chain can easily reach the consumer and pose a serious risk to public health.

## Figures and Tables

**Table 1:** Resistance of *C. jejuni* and *C. coli* isolates of different origins to antibiotics.

Antibiotic	Resistant *Campylobacter* spp. Number of Isolates Depending on Origin/Total Isolates Tested (%)			
	Estonia	Latvia	Lithuania	Human
Nalidixic acid	0/4 (0)	16/16 (100)	26/26 (100)	13/15 (86.7)
Ciprofloxacin	0/4 (0)	16/16 (100)	26/26 (100)	13/15 (86.7)
Tetracycline	0/4 (0)	3/16 (18.8)	20/26 (76.9)	12/15 (80.0)
Streptomycin	0/4 (0)	11/16 (68.8)	11/26 (42.3)	4/15 (26.7)
Erythromycin	0/4 (0)	1/16 (6.3)	3/26 (11.5)	0/15 (0)
Gentamicin	0/4 (0)	0/16 (0)	0/26 (0)	0/15 (0)

**Table 2:** *Campylobacter*-resistant phenotypes.

Antibiotic Resistance Phenotype ^a,b^	*Campylobacter* spp. Number of Strains (%)			
	Estonia (n = 4)	Latvia (n = 16)	Lithuania (n = 26)	Human (n = 15)
Cip/Nal/Tet/Str/Ery	-	-	3 (11.5)	-
Cip/Nal/Tet/Str	-	2 (12.5)	8 (30.8)	4 (26.7)
Cip/Nal/Tet	-	1 (6.2)	9 (34.6)	8 (53.3)
Cip/Nal/Str	-	9 (56.3)	-	-
Cip/Nal/Ery	-	1 (6.2)	-	-
Cip/Nal	-	3 (18.8)	6 (23.1)	1 (6.7)
Resistant to one or more antibiotics	0 (0)	16 (100)	26 (100)	13 (86.7)
Susceptible to all antibiotics	4 (100)	-	-	2 (13.3)
Multidrug resistant ^c^	0 (0)	2 (12.5)	10 (38.5)	4 (26.7)
Total number of tested isolates	4 (100)	16 (100)	26 (100)	15 (100)

^a^ Tested antibiotics: NAL—nalidixic acid; Cip—ciprofloxacin; TET—tetracycline; STR—streptomycin; ERY—erythromycin; GEN—gentamicin. ^b^ The number of resistant isolates was 55. The phenotypes of the antibiotic-resistant isolates were calculated based on 55 isolates. ^c^ Multidrug resistant is defined as strain resistant to three or more unrelated (not belonging to the same class of antibiotics) antimicrobials.

**Table 3:** The minimum inhibitory concentrations of *C. jejuni* and *C. coli* isolates (n = 61).

No. of Isolates	AA ^d^	No. of Isolates with MIC Value (µg/mL) of ^a^										
		0.12	0.25	0.5	1	2	4	8	16	32	64	128
46 ^b^	ERY	-	-	-	40	2	-	-	-	-	-	4 ^(4)^
	CIP	4	-	-	-	1	3	17	21 ^(8)^	-	-	-
	TET	-	-	23	-	-	-	-	1	2	20 ^(16)^	-
	GEN	7	13	20	6	-	-	-	-	-	-	-
	NAL	-	-	-	-	1	-	-	-	3	39 ^(23)^	-
	STR	-	-	6	4	9	3	-	22 ^(20)^	-	-	-
15 ^c^	ERY	-	-	-	15	-	-	-	-	-	-	-
	CIP	2	-	-	-	-	1	7	5 ^(2)^	-	-	-
	TET	-	-	3	-	-	-	-	-	1	11 ^(6)^	-
	GEN	2	2	7	3	1	-	-	-	-	-	-
	NAL	-	-	-	-	-	1	1	-	1	12 ^(6)^	-
	STR	-	-	-	2	5	4	-	4 ^(3)^	-	-	-

^(no)^ Number of *C. jejuni* strains with MIC values exceeding the EUCAMP2 maximum concentration range. ^a^ MIC values for isolates were evaluated according to manufacturer’s instructions (National Veterinary Institute, Uppsala, Sweden). Solid–vertical lines indicate break points between sensitive and resistant isolates for *C. jejuni*, and dashed–vertical lines for *C. coli*, if different from *C. jejuni.* ^b^ Estonian-, Lithuanian- and Latvian-origin broiler chicken meat sampled from Estonian retail in 2018–2019. ^c^ *C. jejuni* and *C. coli* strains of human origin isolated in 2018–2019 in Estonia. ^d^ Antimicrobial agents: NAL—nalidixic acid; Cip—ciprofloxacin; TET—tetracycline; STR—streptomycin; ERY—erythromycin; GEN—gentamicin.

**Table 4:** Comparison of *C. jejuni* and *C. coli* phenotypic and genotypic antibiotic resistance, including mechanisms, patterns, sources and origin.

Antibiotic (Class)	Phenotype/Genotype (n/n)	Mechanism (n)	Pattern (n) ^a^	Source (n)	Country (n: j/c) ^b^
Streptomycin (Aminoglycosides)	16/16	*aadE* (5) ^c^	CIP/NAL/TET/**STR** (5)	Chicken (5)	Lithuania (5: 4j/1c)
		*aadE-Cc* (3)	CIP/NAL/TET/**STR**/ERY (3)	Chicken (3)	Lithuania (3: 3c)
		*aph(3’)-IIIa* (8)	CIP/NAL/**STR** (5)	Chicken (5)	Latvia (5: 5j)
			CIP/NAL/TET/**STR** (3)	Chicken (2)	Latvia (1: 1j)
					Lithuania (1: 1j)
				Human (1)	Estonia (1: 1j)
Erythromycin (Macrolides) ^d^	4/4	*23S* A2075G (4)	CIP/NAL/TET/STR/**ERY** (3)	Chicken (3)	Lithuania (3: 3c)
			CIP/NAL/**ERY** (1)	Chicken (1)	Latvia (1: 1c)
Ciprofloxacin/ Nalidixic acid (Quinolones)	35/35	*gyrA* T86I (35)	**CIP**/**NAL**/TET (12)	Chicken (8)	Lithuania (7: 6j/1c)
					Latvia (1: 1j)
				Human (4)	Estonia (4: 3j/1c)
			**CIP**/**NAL**/TET/STR (8)	Chicken (7)	Lithuania (6: 5j/1c)
					Latvia (1: 1j)
				Human (1)	Estonia (1: 1j)
			**CIP**/**NAL** (6)	Chicken (5)	Lithuania (4: 4j)
					Latvia (1: 1c)
				Human (1)	Estonia (1: 1j)
			**CIP**/**NAL**/STR (5)	Chicken (5)	Latvia (5: 5j)
			**CIP**/**NAL**/TET/STR/ERY (3)	Chicken (3)	Lithuania (3: 3c)
			**CIP**/**NAL**/ERY (1)	Chicken (1)	Latvia (1: 1c)
Tetracycline (Tetracyclines)	23/23	*tetO* (23)	CIP/NAL/**TET** (12)	Chicken (8)	Lithuania (7: 6j/1c)
					Latvia (1: 1j)
				Human (4)	Estonian (4: 3j/1c)
			CIP/NAL/**TET**/STR (8)	Chicken (7)	Lithuania (6: 5j/1c)
					Latvia (1: 1j)
				Human (1)	Estonia (1: 1j)
			CIP/NAL/**TET**/STR/ERY (3)	Chicken (3)	Lithuania (3: 3j)

^a^ Tested antibiotics: NAL—nalidixic acid; CIP—ciprofloxacin; TET—tetracycline; STR—streptomycin; ERY—erythromycin. Bold indicates concurrence between genotypic and phenotypic resistance. ^b^ j—*C. jejuni*; c—*C. coli.* ^c^ One isolate also had the aad9 gene. ^d^ *50S* L22 (A103V) modification was detected in 14 erythromycin MIC-sensitive isolates.

## Data Availability

Molecular analysis, whole-genome sequencing and bioinformatics were performed as described by Tedersoo et al. [25] and all the assembled genomes are accessible from *Campylobacter jejuni/coli* multilocus sequence typing (MLST) database (pubMLST). [https://pubmed.ncbi.nlm.nih.gov/30345391/].
